# Impact of environmental diversity of hunting complexes in the Lublin region on ontogenetic quality indicators in roe deer (*Capreolus capreolus*)

**DOI:** 10.2478/s11756-018-0025-6

**Published:** 2018-03-15

**Authors:** Piotr Czyżowski, Leszek Drozd, Mirosław Karpiński, Katarzyna Tajchman, Małgorzata Goleman, Justyna Wojtaś, Damian Zieliński

**Affiliations:** Department of Ethology and Animal Welfare, University of Life Sciences in Lublin, St. Akademicka 13, 20-950 Lublin, Poland

**Keywords:** roe deer, *Capreolus capreolus*, hunting complex, individual condition

## Abstract

Populations of game are not confined to single ecosystems but function within higher-order units, e.g. ecological landscape. The basis for the establishment of the hunting complexes was the assumption that the existing game hunting grounds, i.e. the basic units implementing game management, are too small and do not cover the natural areas inhabited by game populations. Roe deer are flexible species and easily adapt to various site conditions, so they inhabit many different habitats, from large forest complexes, through small in-field tree stands and shrubs, to treeless grounds and field monocultures. The aim of the study was to determine a possible impact of environmental conditions prevailing in the hunting complexes of the Regional Directorate of State Forests (RDLP in Lublin) on the ontogenetic quality of roe deer. The study was conducted on 518 European roe deer (*Capreolus capreolus*) aged from 4 to 7 years (379 bucks and 139 does) harvested within hunting seasons 2010/2011–2013/2014. The results have shown that animals originating from areas with greater forest cover and denser stands are characterised by lower values of the mean ontogenetic quality parameters (carcase weight, kidney fat index, chest girth, weight of antlers) in comparison with animals from typical agricultural areas with fragmented forest complexes. These results indicate that, even in the case of such a eurytopic species as the roe deer, the ontogenetic quality differs between individual hunting complexes. The study has proved that strategies for hunting management of the roe deer should take into account the impact of the landscape structure, which provides a rationale behind creation of hunting complexes.

## Introduction

In order to ensure rational management of game and maintenance of their biodiversity, hunting complexes have been established in all Regional Directorates of State Forests (RLPD). These areas were created by combining several neighboring forest districts with similar natural and physiographic environmental conditions, which determine a similar way of conducting game management.

The basis for the establishment of the hunting complexes was the assumption that the existing game hunting grounds, i.e. the basic units implementing game management, are too small and do not cover the natural areas inhabited by game populations (Raczyński et al. 2011). Populations of game animals, in particular those of large and mobile species, are not confined to single ecosystems but function within higher-order units, e.g. ecological landscape. Management of the deer and moose extends considerably beyond the area of game hunting districts; they have been identified as a major species present in hunting complexes predominated by forest hunting grounds. The roe deer (*Capreolus capreolus* Linnaeus 1758) have been classified as a major species in forest hunting complexes; however, the eurytopicity of the species is disregarded (Saïd and Servanty 2005).

The aim of the study was to determine the effect of different environmental conditions prevailing in the RDLP hunting complexes on the ontogenetic quality of the roe reflected by such indicators as the carcase weight, kidney fat index (KFI), chest girth, weight of antlers, and creatinine level.

## Study area

The roe deer originated from the hunting complexes of the RLPD in Lublin. The areas differ from each other in environmental factors, e.g. the agricultural production quality index (the mean score for the communes covered by each region) (Witek et al. 1993), forest fragmentation index - a ratio of the total length of forest boundaries (km) to the total area of forest complexes (km^2^) (Czyżowski et al. 2011), forest cover (%), and percent proportion of forest habitats according to the fertility groups (coniferous habitats, mixed coniferous habitats, mixed forest habitats, and forest habitats) (data obtained from RDLP in Lublin) (Table 1).

**Table 1:** Percentage of selected environmental factors in different regions of Regional Directorate of State Forests in Lublin

Hunting complexes	Indicator of the quality of agricultural production (pts)	Forest cover (%)	Forest fragmentation (km/km^2^)	Coniferous forest (%)	Mixed coniferous forest (%)	Mixed deciduous forest (%)	Deciduous forest (%)
Sandomierska Primeval Forest	64.4	45.8	1.3	41.9	31.7	16.9	8.5
Roztocze and Solska Primeval Forest	79.7	27.4	2.7	24.2	17.7	18.6	34.5
Łęczna-Włodawa Lakeland	53.8	45.5	1.6	40.7	27.8	18.6	10.9
Lublin Region	82.1	13.2	3.3	4.5	8.4	18.2	68.5
Puławy-Lubartów Region	65.5	23.1	2.0	17.8	22.7	42.2	12.4
Janowskie Forest	62.2	46.6	1.0	42.3	39.1	15.9	1.6

## Material and methods

The study material included 518 European roe deer (*C. capreolus*) individuals aged 4–7 years (379 roebucks and 139 does) harvested within hunting seasons: 2010/2011–2013/2014 in accordance with the applicable Rules of Individual and Population Game Animal Selection in Poland (Annex to Resolution No. 57/2005 of February 22, 2005). Measurements of the carcase weight (kg), KFI, trophy and skull weight (g), chest girth (cm), and creatinine levels (mg dl^−1^) were carried out. Kidneys dissected from the carcasses were weighed with an accuracy of 1 g. Next, the kidney fat was removed and the kidneys were weighed again. Based on the measurements results, KFI was calculated as a quotient of the kidney weight with fat and without fat (Riney 1955). The carcass weight (roebuck body without the head) was measured with an accuracy of 0.5 kg. The chest girth was measured around the trunk behind the ulnar tubers using a zoometric tape. The creatinine levels were determined in blood collected into test tubes with a coagulant immediately after culling the animals.

The serum creatinine levels were determined using the spectrophotometric method in the Hematological and Serological Laboratory at the Infectious Diseases Clinic, Department of Epizootiology and Clinic of Infectious Diseases, Faculty of Veterinary Medicine in Lublin. The age of the culled animals was identified based on the degree of tooth wear (Lochman 1987).

Statistical analysis of the research results was carried out with the use of the Statistica 13.1 PL package. The normality of distributions of the analysed parameters was assessed with the Shapiro-Wilk test. Since the distributions of the dependent variables significantly diverged from normality, nonparametric (rank) tests were employed to analyse the significance of the differences between these distributions. A non-parametric analysis of variance, i.e. Kruskal-Wallis ANOVA, was used for comparison of the mean values between multiple groups (hunting complexes). The description of the distributions was based on the measures of the position of the mean value, i.e. the median and quartiles. In the case of comparison of multiple groups and significant differences between these groups, the results of the multiple comparison tests have the so-called letter marking. Groups denoted with the same letter are not significantly different from each other (they represent the same homogeneous group), whereas groups that are not marked with the same letter differ significantly.

## Results

The analysis of the differences in the mean carcass weight between animals from the different hunting complexes (Table 2) revealed that roebucks from the Puławy-Lubartów region, Sandomierska Primeval Forest, Roztocze, and Solska Primeval Forest were characterised by the highest carcass weight. The lowest carcass weight was determined for roebucks from the Janowskie Forest region, and the difference was statistically significant (ANOVA Kruskal-Wallis rank test rang, χ^2^ = 24.23; *p* = 0.0001).

**Table 2:** Values of the parameters analysed in *Capreolus capreolus* bucks (median, Q25-Q75, n) in particular hunting complexes

	Hunting complexes					
	Sandomierska Primeval Forest	Roztocze and Solska Primeval Forest	Lublin Region	Puławy-Lubartów Region	Janowskie Forest	*p*
Carcass weight	19.0^a^ (17.0–20.0) 122	19.0^a^ (17.0–20.0) 77	18.0 (17.0–19.0) 53	19.5^a^ (17.0–21.0) 44	17.0^b^ (15.0–18.0) 83	0.0001
Kidney Fat Index (KFI)	1.231^a^ (1.13–1.33) 122	1.159 (1.10–1.24) 77	1.167 (1.11–1.22) 53	1.180^a^ (1.11–1.46) 44	1.147^b^ (1.09–1.24) 83	0.0026
Weight of antlers	345^a^ (297–420) 59	348^a^ (318–424) 77	436^a^ (302–484) 31	308^a^ (220–360) 25	167^b^ (147–200) 15	0.0008
Chest girth	70.0 (68–74) 38	73.0^a^ (71–76) 19	70.0 (68–71) 15	79.5^a^ (75–85) 18	63.5^b^ (60–72) 14	0.0000
Creatinine level	1.73 (1.5–2.1) 26	1.56 (1.4–1.9) 30	1.60 (1.4–1.7) 9	1.75 (1.6–1.9) 13	1.69 (1.4–2.0) 16	0.6613

^a^, ^b^ - The values in the rows marked with different letters within each hunting ground differ significantly at *p* ≤ 0.01 (rank order multiple comparison test)

The distribution of the mean KFI was similar, i.e. the highest mean KFI value was noted for roebucks from Sandomierska Primeval Forest and the Puławy-Lubartów region, whereas the lowest value was reported from the Janowskie Forest region; the difference was statistically significant (χ ^2^ = 16.33; *p* = 0.0026).

The highest average weight of antlers was determined in the group of roebucks from the Lublin region, while the animals from the Janowskie Forest region were characterised by the lowest value, and the difference was statistically significant, in comparison with the roebucks from all the analysed regions (χ ^2^ = 18.84; *p* = 0.0008).

The distribution of the mean values of the chest girth was similar to that of the other analysed parameters. The highest values were calculated for roebucks from the Puławy-Lubartów region, Roztocze, and Solska Primeval Forest, and the lowest values were reported from the Janowskie Forest region (χ ^2^ = 28.28; *p* = 0.0000).

The highest values of the mean creatinine levels were noted in animals from Solska Primeval Forest and the Lublin region and the lowest values were reported from Roztocze and Solska Primeval Forest; however, the differences were not statistically significant (χ ^2^ = 2.41; *p* = 0.6613).

The highest mean values of carcass weight of does (Table 3) have been noted in Łęczna-Włodawa Lakeland and the Puławy-Lubartów region. In turn the lowest mean values of carcass weight were noted for does from Roztocze and Solska Primeval Forest regions, and the difference was statistically significant (χ^2^ = 16.11; *p* = 0.0029). As revealed by the analysis of the differences in the KFI values noted for does, the highest mean value of this parameter was noted in Sandomierska Primeval Forest and the Lublin region and the lowest value was determined for Łęczna-Włodawa Lakeland (χ^2^ = 14.26; *p* = 0.0065). The highest values of the chest girth were found in the doe group from Łęczna-Włodawa Lakeland, and the difference was statistically significant in comparison with the values noted for does from Sandomierska Primeval Forest, Roztocze, and Solska Primeval Forest (χ^2^ = 18.97; *p* = 0.0008). The highest mean values of the creatinine level were noted in animals from Łęczna-Włodawa Lakeland and the lowest values were reported for those from Sandomierska Primeval Forest; however, this difference was not statistically significant (χ ^2^ = 9.30; *p* = 0.0550).

**Table 3:** Values of the parameters analysed in *Capreolus capreolus* does (median, Q25-Q75, n) in particular hunting complexes

	Hunting complexes					
	Sandomierska Primeval Forest	Roztocze and Solska Primeval Forest	Łęczna-Włodawa Lakeland	Lublin Region	Puławy-Lubartów Region	*p*
Carcass weight	17.0 (16.0–18.0) 9	17.0^a^ (16.0–18.0) 36	19.0 (17.0–20.0) 38	19.0 (17.0–20.0) 25	19.0^b^ (18.0–20.0) 31	0.0029
Kidney Fat Index (KFI)	1.364^a^ (1.33–1.73) 9	1.273 (1.21–1.34) 36	1.348 (1.13–1.57) 38	1.151^b^ (1.11–1.18) 25	1.222 (1.13–1.41) 31	0.0065
Weight of antlers	64.0^a^ (60–76) 7	70.0^a^ (64–72) 16	73.5^b^ (72–77) 28	71.0 (69–72) 18	72.0 (72–77) 13	0.0008
Chest girth	1.60 (1.5–1.7) 4	1.63 (1.5–1.8) 8	2.05 (1.7–2.1) 3	1.89 (1.9–1.9) 5	1.71 (1.6–2.1) 10	0.0550

^a^, ^b^ - The values in the rows marked with different letters within each hunting ground differ significantly at *p* ≤ 0.01 (rank order multiple comparison test)

## Discussion

Environmental conditions prevailing in habitats have a direct effect on the physical status of animals. Body weight, which reflects the fitness of an individual and determines its survivability and reproductive success, is the basic criterion in assessment of the ontogenetic quality of wild-living animals (McElligott et al. 2001; Toïgo et al. 2006). Parameters of the physical status of an individual can fluctuate seasonally and depend on nutrient deficiency and the physiological status of the organism, e.g. parasitic diseases (Sams et al. 1998). Therefore, the use of multiple parameters for description of the ontogenetic quality of roe deer is advisable.

The present study demonstrated that roebucks from the hunting grounds in the Janowskie Forest region were characterised by significantly lower mean values of physical status parameters than animals from the other analysed regions. This area is characterised by the largest forest cover of the Lubelskie region (Table 1). They include the two following habitats: coniferous forest and fresh broadleaved forest. Lower weight of roe deer was observed in non-fertile forest habitats. This phenomenon can be explained by the fact that they provide less easy-to-digest and high calorie food in the form of herbaceous plants needed for goats during the reproductive period (Jackson 1980; Kałuziński 1982; Mysterud et al. 1999; Heinze et al. 2011). Similar significantly lower differences in the roe deer weight were reported from poor forest habitats by other authors (Pettorelli et al. 2002; Wajdzik et al. 2016). Additionally, the large forest cover in the region and the highest compactness of the forest complexes are accompanied by poor quality of agricultural soils and, as indicated by other investigators, the roe deer prefers a mosaic of habitats providing diverse food sources, which meet the nutrient demand more efficiently (Tufto et al. 1996). In other studies (Janiszewski et al. 2009; Petelis and Brazaitis 2003; Flis 2011; Kamieniarz 2013), roebucks from agricultural ecosystems were characterised by higher carcass weight in comparison with individuals living in typical forest areas, which was associated with increased availability of highly nutritious food.

The lowest mean value of the KFI noted in the Janowskie Forest region confirms the lower fitness of roebucks living in this typical forest area. The amount of fat deposited around the internal organs is a direct indication whether the animal lives in favourable environmental and feeding conditions, which is reflected in its fitness (Serrano et al. 2008). The roebucks from the Janowskie Forest region were characterised by the lowest antler weight in comparison with the animals from the other hunting complexes. The heaviest antler was found in the Lublin region, which has the lowest forest cover and the highest forest fragmentation index. This region has a typically agricultural character, which is indicated by the high agricultural production quality index. Therefore, it ensures availability of high calorie food, which may have a direct effect on antler quality (Kruuk et al. 2002).

The roebucks from the Janowskie Forest region were characterised by the lowest mean value of the chest girth in comparison with animals from regions characterised by lower forestation levels and higher forest fragmentation indices. The increase in the biometric parameters of the chest is highly correlated with body weight (Watkins et al. 1991) and is significantly correlated with physiological traits, in particular with the function of the cardiovascular system (Egstrom et al. 1966), which exerts a direct effect on the physical condition and fitness of the organism.

In the present study, the level of serum creatinine was also used for comparison of the ontogenetic quality of the roe deer from the analysed hunting complexes. In healthy animals, higher serum creatinine levels are associated with higher muscle weight and increased physical activity, which reflects high cardiac efficiency and good physical fitness of the animal (Baxmann et al. 2008).

The analysis of the mean values of the parameters characterising roebucks from the other hunting complexes demonstrated their high values in regions that differed substantially in terms of the environmental factors. An example is the high carcass weight of animals from Sandomierska Primeval Forest, Roztocze, and Solska Primeval Forest as well as the high KFI values in Sandomierska Primeval Forest and the Puławy-Lubartów region. This confirms the findings reported by many authors (Hewison et al. 2001; Pettorelli et al. 2001; Morellet et al. 2011; Kamieniarz 2013) that the roe deer is an exceptionally flexible and eurytopic species among *Cervidae*; therefore, it functions well in compact forest complexes and in typical agricultural landscape (Kulak and Wajdzik 2009; Wajdzik et al. 2007).

Does with the highest body weight originated from typical agricultural areas (Lublin and Puławy-Lubartów regions) and from regions with large forest cover (Łęczna-Włodawa Lakeland). The distribution of the mean KFI values differed. Does originating from areas with large forest cover and predominance of coniferous habitats (Sandomierska Primeval Forest and Łęczna-Włodawa Lakeland) were characterised by the highest KFI values. The does were harvested in an autumn-winter period and, as indicated by other authors (Bobek et al. 2016; Janiszewski and Szczepański 2001), coniferous habitats are the main refuges for *Cervidae* during wintertime, as they provide better feed and preserve conditions than mixed and deciduous forests. *Vaccinium* plants collected in these habitats are an important element in the winter diet for roe deer (Cederlund et al. 1980; Mysterud and Østbye 1995).

## Conclusions

The investigations have shown that, even in the case of such a eurytopic species as the roe deer, the ontogenetic quality differs between hunting complexes, which is evidenced by the differences in the mean values of the ontogenetic quality parameters. The study has proved that strategies for hunting management of the roe deer should take into account the impact of the landscape structure, which provides a rationale behind creation of hunting complexes. This type of research is consistent with the implementation of a large-scale hunting design and can be helpful in establishment of precise principles of population and individual selection in hunting complexes.
